# Depression and subjective well-being in older adults living in low-density areas during the COVID-19 pandemic: the role of social participation and social support

**DOI:** 10.3389/fpubh.2026.1771874

**Published:** 2026-06-24

**Authors:** Rita Francisco, Samuel Domingos, Nádia Cruz, Rui Gaspar, Marta Pedro, Cristina Godinho

**Affiliations:** 1 School of Human Sciences, Universidade Católica Portuguesa, Lisboa, Portugal; 2 HEI-Lab: Digital Human-Environment Interaction Labs, Universidade Lusófona, Lisboa, Portugal; 3 NOVA National School of Public Health, Public Health Research Centre, Comprehensive Health Research Center, CHRC, NOVA University Lisbon, Lisboa, Portugal

**Keywords:** COVID-19, depression, mediation analysis, older adults, social participation, social support, subjective well-being

## Abstract

**Introduction:**

Participation in social activities contributes to enhanced well-being, reduced rates of comorbidities, and diminished feelings of depression among older adults. However, the COVID-19 pandemic has altered social interaction in many ways, and its impacts on the well-being and mental health of older adults, particularly those living in more isolated regions, are still poorly understood. This cross-sectional study intends to investigate the relationship between social participation, social support, subjective well-being, and depression among older adults residing in a region with low population density, as well as to explore the role of social support mediating the relation between social participation and subjective well-being and depression, in the context of restrictions imposed during COVID-19 pandemic.

**Methods:**

A total of 324 older adults (M = 75.11; SD = 6.89), living in the Alentejo region (Portugal), randomly selected, answered a questionnaire which included measures about the perception of social participation and social support during the most critical periods of COVID-19 pandemic, subjective well-being, depression, and sociodemographic and general health data.

**Results:**

Correlation analysis showed that older adults’ social participation during the COVID-19 pandemic was associated with higher social support, higher subjective well-being, and lower levels of depressive symptoms. The mediation analysis showed that social support fully mediates the relationship between social participation and subjective well-being, and between social participation and depression.

**Discussion:**

The COVID-19 context may have constrained the expected direct benefits of social participation (e.g., due to social distancing or digital barriers) on well-being and mental health, making social support the primary pathway. The study highlights the importance of making older adults’ social participation relevant and meaningful, reinforcing the role it can play in identifying people who are more vulnerable to depression and strengthening social support networks, with the aim of supporting older people who are more isolated.

## 1 Introduction

In late 2019, initial reports surfaced regarding the outbreak of a new virus, SARS-CoV-2, whose rapid transmission would soon escalate into a global health crisis. In early March 2020, the World Health Organization (WHO) officially declared COVID-19 a global pandemic, prompting the implementation of urgent and drastic measures aimed at containing the virus’s transmission (1, 2). On 18 March 2020, the Portuguese government declared a state of emergency in response to the COVID-19 pandemic. A series of public health directives and official guidelines were issued, most notably the implementation of strict mobility restrictions, and the encouragement of home confinement—which later became mandatory for a period of time—particularly for high-risk groups such as the older adults population. In addition to mobility restrictions, a wide range of establishments and institutions—including schools, recreational centres, and cultural spaces—were closed, and visits to nursing homes and hospitals were restricted (3). As the emergency escalated, a general atmosphere of fear and instability emerged, fuelled by the uncertainty surrounding a global pandemic caused by a novel virus, and further intensified by the overwhelming flow of information, often inconsistent or misleading. While the measures imposed by the government fell within a set of guidelines aimed at disease prevention (4), they also led to significant impacts on individuals’ well-being, further exacerbated by other factors related to the severity of the situation and the prevailing context of uncertainty (5).

As Aristotle famously stated, “man is by nature a social animal.” Forming meaningful connections, living in community, and engaging in shared experiences are essential to human life, with the quality of these relationships being a key determinant of individual well-being. Two theoretical approaches are relevant to explain how relational resources impact mental health and protect individuals during times of adversity: social capital theory and stress-buffering model. Social capital refers to the structure and quality of social relationships, from which individuals, social groups and society may benefit [e.g., (6, 7)]. Although there is no consensus in the literature regarding the definition of social capital (8), most scholars agree that it is a multidimensional construct, developed by trust, reciprocity, and mutual aid, that refers to mutual relationships with the community and the resources that can be gained through this network [e.g., (9)]. Social capital is theorised to protect against mental health problems in two ways: (1) by providing strong social ties that foster and sustain healthy affective, cognitive, and emotional states, or (2) during periods of adversity, by acting as a buffer against stressors that can have a negative impact on health (10). The classic formulation of the stress-buffering model proposes two pathways through which social support can influence health and psychological adjustment: (1) a main effect, according to which social support is associated with better psychological and social functioning, regardless of the level of stress experienced, or (2) a buffering effect, in which social support can reduce the impact of stressful events on mental health and well-being. This effect can occur at different moments in the process: before the stress response, by contributing to the situation being perceived as less threatening, and afterwards in the appraisal of the stressor, by helping to cope with adversity, namely through emotional regulation and the mobilisation of resources (11, 12). Together, these approaches help to explain how relational resources protect individuals during times of adversity, and how these dynamics transform over time.

In the context of the pandemic, the chronic or recurring stressors to which older people are exposed—such as grief, the loss or readjustment of social roles, chronic illness, comorbidities, functional decline, or isolation (13, 14)—may have been intensified by the reduction of social contact, the disruption of routines, and the decrease in opportunities for social participation (15). This context may assume particular relevance among older people living in rural and low-density areas, where access to services, transport, healthcare, and community resources may be more limited (16–18). In these contexts, relational resources become especially important when adaptive demands accumulate, potentially functioning as a source of protection against adverse experiences, loneliness, isolation, and depression. In this sense, the present study draws on social capital theory and the stress-buffering model to conceptualise social relationships as a protective resource, capable of helping to understand the relationship between social participation, social support, and indicators of mental health and well-being in older adults in times of adversity.

Social participation—defined as the active engagement in activities that foster interaction with other members of the community, in settings such as religious, cultural, recreational, political, or sports organisations (15)—is associated with a range of protective factors for health, particularly among the older adults population [e.g., (19)]. For example, older people’ participation in social activities has been shown to contribute to enhanced well-being, reduced rates of comorbidities, and diminished feelings of loneliness and isolation (15). Moreover, research indicates that social engagement may mitigate cognitive decline and enhance quality of life, by stimulating cognitive functions and promoting physical activity, which are vital for maintaining health (15, 20). Participation in such activities, in addition to fostering greater emotional resilience and supporting active and healthy ageing, also promotes self-esteem and strengthens interpersonal bonds, resulting in a solid social support network which, in itself, serves as a protective factor against depression (15, 19, 21).

Social support, in turn, is a critical determinant of emotional resilience, health, and well-being. It is understood as the set of social and emotional resources accessible through an individual’s network, which may include family, friends, neighbours, and institutions (22, 23). Research has shown that social support is a significant contributor to well-being, as it enhances individuals’ ability to adapt to adversity and is also associated with greater longevity (24). In times of crisis, such as the COVID-19 pandemic, social support plays a particularly vital role, as its presence fosters a sense of safety, comfort, and belonging, all of which contribute to the preservation of well-being (21, 22, 25). Empirical evidence suggests that older adults with more robust support networks face a reduced risk of cardiovascular illness, are less likely to experience psychological disorders, and exhibit stronger immune responses (26). In contrast, the absence of social support increases the risk of mental health conditions such as anxiety and depression (27).

Social participation and support have been consistently identified by research as crucial determinants of overall well-being and of both physical and mental health (28–35). Nevertheless, the specific relationship between them is not yet entirely clear, and has been less extensively studied in the older adults population. For example, the study by Li et al. (36), conducted in China, identified social support as a mediator of the influence of social participation on lower levels of depressive symptoms among older adults. However, although greater social participation was associated with greater life satisfaction, social support was not.

The challenges posed by the COVID-19 pandemic were particularly heightened for the older adults population. For instance, the perception of social isolation, feelings of loneliness, reduced or absent interpersonal interactions, decreased participation in recreational and social activities, and the enforcement of physical distancing measures during the pandemic, have all been identified as significant predictors of declines in subjective well-being, deterioration of physical and mental health, and increased mortality and morbidity among the older population (35, 37–46). These challenges may have been particularly evident in regions where geographic dispersion and population ageing already posed barriers to maintaining social contact. In these areas, the physical distance from family members, who often live in other regions or abroad, made access to immediate support more difficult.

Due to the restrictions imposed and the increased difficulty in accessing and mastering technological tools (47), many older adults were unable to see their family and friends for extended periods. This situation resulted in the deterioration of both cognitive and emotional health, which, in turn, led to the development of anxiety and depression symptoms among institutionalised and non-institutionalised older adults, as a result of the absence of affective stimuli and meaningful social interactions (25, 48, 49). The closure of day centres and institutions providing access to cultural, religious, and sports practices severely restricted opportunities for older adults to engage with their communities (15, 49).

While research indicates that some degree of social participation persisted among older adults during the COVID-19 pandemic through digital means, they were not equally accessible to all older adults. Use of these means was particularly low for people with limited digital skills or reduced access to technology, like those with low education levels or residing in rural settings (50, 51). On the other hand, it is widely recognised that physical touch and proximity are fundamental components of emotional and social well-being, with a profound impact on the quality of individuals’ relationships. In the absence of this type of contact, individuals tend to experience heightened feelings of isolation and loneliness, often accompanied by distress, anxiety, and sadness. Accordingly, the absence of physical closeness and touch may impair individuals’ perceived social support, weaken their sense of belonging, and increase their susceptibility to emotional and psychological challenges (21). In this regard, the restriction of in-person contacts substantially diminished access to support networks, increasing the psychological vulnerability of older adults to the effects of the restrictions. Particular emphasis is placed on the effects of physical distancing, social isolation, feelings of loneliness, the shrinking of social networks and the decline in interaction quality, as well as the lack of social support, on depression levels and mortality rates among the older adults population (43, 52).

### 1.1 The current study

Given the importance of social capital—in particular social participation and social support—for subjective well-being and depression among older adults, and considering the constraints imposed during the COVID-19 pandemic, which substantially limited opportunities for social interactions and created distance from support networks, there is a pressing need to explore the interplay between these variables during periods of adversity and evaluate the extent of their implications. Furthermore, it is important to investigate it among older adults living in geographical areas with a high proportion of older people and low population density, such as the Alentejo region in Portugal. The Alentejo is one of the regions with the oldest population in Portugal, home to the highest number of isolated older people and the highest suicide rate. It is therefore an ideal setting in which to explore the links between the isolation caused by the COVID-19 pandemic, social capital, and the well-being of these communities. The results can be useful for developing future strategies to mitigate their impact, especially in future situations similar to the COVID-19 pandemic.

Thus, the present study aims to: (a) explore the relationship between social participation, social support, subjective well-being and depression, among older adults living in low-density areas; and (b) test whether social support mediates the relationship between social participation and subjective wellbeing (model 1) and between social participation and depression (model 2).

Based on the theoretical framework and empirical studies presented above, we propose the following hypotheses: (1) increased levels of social participation would be associated with higher social support, higher subjective well-being and lower levels of depressive symptoms among older adults; (2) social support would partially mediate the relationship between older adults’ social participation and subjective well-being, considering that higher social participation would also influence directly higher levels of subjective well-being; and (3) social support would partially mediate the relationship between older adults’ social participation and depression, considering that poorer social participation would also influence directly higher levels of depression.

## 2 Materials and methods

### 2.1 Participants

A total of 324 older adults aged 65 to 92 years (M = 75.11; SD = 6.89), living in the Alentejo region (Portugal), participated in the study. The majority were female (67.9%). Of the total number of participants, 50.9% lived in rural areas (most towns with less than 2,000 inhabitants) and the rest in urban areas (with more than 5,000 inhabitants). Most participants indicated their marital status as married/marital partnership (53.1%) or widowed (37.7%), with the remainder indicating that they were divorced (5.6%) or single (3.7%). Of the total sample, 35.2% lived alone.

In general, the participants’ education level was low, with the majority reporting only having attended or completed the first cycle of basic education, i.e., up to 4 years of schooling (52.8%). Regarding the assessment of socio-economic status, a high proportion indicated that their financial situation was sufficient for the needs of the household (43.8%), although 38.9% reported their financial situation as difficult or very difficult.

As for self-perception of health status, many rated their health as “neither good nor bad” (47.5%), 34.3% rated it as “good,” and 15.1% as “bad.” Of all the participants, 50.6% reported having a chronic illness (e.g., diabetes, hypertension, cancer, depression). With regards to COVID-19, only 36.7% reported having had COVID-19 at the time of the study. In addition, 42.6% of participants reported the death, by any cause of death, of at least a close one during that period.

### 2.2 Measures

The questionnaire that was applied included the following measures:

#### 2.2.1 Social participation

Social participation levels were assessed using the Portuguese version of the short version of the Australian Community Participation Questionnaire (ACPQ-15) (53–55), which is grounded in a conceptualization of social participation that includes informal social connectivity, civic engagement, and political participation. The introductory instruction of this instrument was adapted for the present study, thereby enabling the evaluation of participants’ perceived social participation during the most critical phases of the pandemic. It consists of 15 items (e.g., “During the most critical periods of the pandemic, my neighbours would share their news with me, or I would share mine with them”), rated on a 7-point scale (from 1 = “Never or almost never” to 7 = “Always or almost always”), enabling the evaluation of seven dimensions of social participation: (1) Contact with household members; (2) Contact with extended family; (3) Contact with friends; (4) Contact with neighbours; (5) Religious practice; (6) Participation in organised community activities; and (7) Active interest in current affairs. The raw score is determined by summing the responses to all 15 items, yielding a total score ranging from a minimum of 15 to a maximum of 105 points. Higher scores indicate greater levels of perceived social participation during the COVID-19 pandemic. The scale exhibited moderate internal consistency (Cronbach’s *α* = 0.80), slightly lower than that found for the Portuguese population in its original version [α = 0.87; (55)].

#### 2.2.2 Social support

Social support levels were assessed using the Portuguese version of the Multidimensional Scale of Perceived Social Support [MSPSS; (56, 57)]. The original instruction was adapted for the present study, thereby enabling the assessment of perceived social support from family, friends, and significant others since the onset of the COVID-19 pandemic. This instrument, divided into three subscales (family, friends, and significant others), consists of 12 items (e.g., “I can count on my friends when something goes wrong”), rated on a 7-point scale (from 1 = “Strongly disagree” to 7 = “Strongly agree”). A total score is obtained by summing the responses to the 12 items, ranging from 12 to 84 points (56, 57). Higher scores indicate greater levels of perceived social support since the onset of the COVID-19 pandemic. The measure demonstrated a high level of internal consistency (Cronbach’s *α* = 0.92), similar to that found for the Portuguese population in its original version (α = 0.94; (56)).

#### 2.2.3 Subjective well-being

Subjective well-being was assessed using the Portuguese translation of the WHO-5 Well-Being Index (58, 59), a widely recognised and extensively validated tool for assessing psychological well-being (60, 61). This instrument comprises five unidimensional items reflecting positive feelings of well-being (e.g., “Over the past 2 weeks, I have felt cheerful and in good spirits”), rated on a six-point Likert scale ranging from 0 (“At no time”) to 5 (“All of the time”). The total raw score is calculated by summing responses across all five items, yielding a score between 0 and 25, with higher scores indicating better levels of subjective well-being. The measure demonstrated a high level of internal consistency (Cronbach’s *α* = 0.91).

#### 2.2.4 Depression

Depression levels were assessed using the Depression subscale of the Portuguese version of the Depression, Anxiety and Stress Scales [DASS; (62, 63)]. This instrument enables a subjective evaluation of depressive symptomatology and encompasses indicators such as dysphoria, devaluation of life, self-depreciation, lack of interest or involvement, anhedonia, and inertia. The subscale consists of seven items (e.g., “Over the past week, I felt down-hearted and blue”) that reflect negative emotional symptoms. Each item is rated on a 4-point Likert scale, ranging from 0 (“Did not apply to me at all”) to 3 (“Applied to me most of the time”). The total score is obtained by summing the responses to the seven items and multiplying the result by two, yielding a final score ranging from 0 to 42 (76). Higher scores indicate more negative affective states and are associated with greater levels or likelihood of depression. The measure demonstrated good internal consistency (Cronbach’s α = 0.89), slightly higher than the version adapted for the Portuguese population [α = 0.85; (63)].

#### 2.2.5 Sociodemographic and general health data

Some participants’ sociodemographic data were collected, including gender, age, marital status, education, place of residence, number of people in the household, socioeconomic status, as well as general data about participants’ health, such as self-perception of health status (“How do you rate your health?,” rated on a 5-point Likert scale from “very bad” to “very good”) and COVID-19 indicators (e.g., having had COVID-19 infection).

### 2.3 Data collection procedures

After the project was approved by the University’s Ethics Committee (ref. CETCH2022-21), data collection was carried out by the Centre for Studies and Opinion Polls of the Portuguese Catholic University (CESOP) between October 15 and November 5, 2022, in the Alentejo region (Portugal). Sampling followed a random procedure for selecting participants based on parishes and households. This also ensured the inclusion of participants (i.e., subsamples) residing in the different sub-regions of Alentejo (Lezíria do Tejo; Alentejo Litoral; Alto Alentejo; Alentejo Central; Baixo Alentejo). According to statistics for Portugal, a sample size of 300 participants would be sufficient to provide a representative sample of the population aged over 65 in this region. A group of independent interviewers from CESOP, trained in data collection methodology and in applying the study’s research protocol, collected all data using a “door-to-door” format, using a Computer-Assisted Personal Interviews (CAPI) method. These interviewers approached the randomly selected participants in their households. Those who gave their informed consent to participate (57% acceptance rate) answered the questions put to them by the interviewer, who recorded their responses on a tablet.

### 2.4 Data analysis procedure

Descriptive statistical analysis procedures were first carried out to characterise sample sociodemographic characteristics. With the aim of exploring the relationship between social participation, social support, subjective well-being and depression, and also with self-perception of health status and sociodemographic variables, Pearson correlations were calculated. To explore the role of social support mediating the relation between social participation and subjective well-being (model 1) and between social participation and depression (model 2), two separate mediation analysis were conducted using the PROCESS Macro for SPSS (64). In these analyses we controlled for perceived health, age, gender (being female vs. male), and living status (alone vs. with others). Indirect effects were estimated using the PROCESS Macro and tested for significance using bias-corrected bootstrap confidence intervals (5,000 samples). An indirect effect was considered significant when the confidence interval did not include zero. All analyses were conducted using the IBM SPSS statistical analysis software. The verification of assumptions (e.g., normality; absence of multicollinearity) required for conducting the reported statistical analysis were carried out in accordance with the procedures and guidelines proposed by Marôco (65, 66) and Tabachnick and Fidell (67). A significance level of *α* = 0.05 was considered for all analyses.

## 3 Results

### 3.1 Descriptive statistics

Table 1 presents descriptive statistics for social participation (contact with household members; contact with extended family; contact with friends; contact with neighbours; religious practice; participation in community activities; active interest in current affairs), social support (family; friends; significant others), subjective well-being, and depression. Results indicate moderately low levels of social participation during the COVID-19 pandemic. Contact with household members and with extended family were the dimensions of social participation rated higher by the participants, while participation in community activities and religious practice were lower. Levels of social support during the COVID-19 pandemic reported by the participants where moderately high, with support from significant others being rated as the highest form of social support reported by the participants and support from friends the lowest. Participants reported moderately high levels of subjective well-being, and low levels of depressive symptomatology.

**Table 1:** Descriptive statistics for social participation, social support, subjective well-being, and depression.

Variable	Range	*M*	*SD*	Range*_Raw Score_*	*M_Raw Score_*	*SD_Raw Score_*
Social participation	1–7	2.85	0.91	15–105	41.74	14.09
Contact with household members	1–7	5.71	1.84		9.63	5.35
Contact with extended family	1–7	3.79	2.01		7.36	4.12
Contact with friends	1–7	2.73	1.42		5.41	2.88
Contact with neighbours	1–7	3.07	1.54		6.09	3.07
Religious practice	1–7	2.04	1.51		4.01	2.92
Participation in community activities	1–7	1.55	1.05		4.63	3.14
Active interest in current affairs	1–7	2.31	1.43		4.60	2.88
Social support	1–7	5.38	0.97	12–84	63.70	11.94
Family	1–7	5.51	1.12		21.76	4.67
Friends	1–7	4.98	1.23		19.69	5.23
Significant others	1–7	5.62	1.15		22.26	4.67
Subjective well-being				0–25	14.85	7.50
Depression				0–42	8.97	9.55

### 3.2 Correlations

Table 2 shows correlations between social participation, social support, subjective well-being, depression, health status, and sociodemographic variables. Social participation shows a weak significant positive correlation with subjective well-being, and a moderate significant positive correlation with social support. Social support also shows a weak significant positive correlation with subjective well-being. Depression presents moderate significant negative correlations with subjective well-being and social support, and a weak significant negative correlation with social participation.

**Table 2:** Correlations between social participation, social support, subjective well-being, depression, perceived health status, and sociodemographic variables.

Variable	1	2	3	4	5	6	7
1. Social participation	–						
2. Social support	0.308**	–					
3. Subjective well-being	0.212**	0.229**	–				
4. Depression	−0.263**	−0.379**	−0.554**	–			
5. Health status	0.215***	0.244***	0.388***	−0.353***	–		
6. Age	−0.092	−0.079	−0.153**	0.234***	−0.090	–	
7. Gender^a^	−0.113*	−0.096	−0.138*	0.222***	−0.064	0.055	–
8. Living status^b^	−0.317***	−0.212***	−0.057	0.206***	−0.052	0.202***	0.257***

*** p < 0.001, ** *p* < 0.01, * *p <* 0.05; ^a^ Dummy variable (0 = male, 1 = female); ^b^ Dummy variable (0 = living with other people, 1 = living alone).

Self-perceived health status shows weak significant positive correlations with social participation and social support, moderate significant positive correlation with subjective well-being, and a moderate significant negative correlation with depression. Both age and gender (female) present weak significant negative correlations with subjective well-being, and positive with depression. The living status (living alone) presents moderate significant negative correlation with social participation, weak significant negative correlation with social support, and weak significant positive correlation with depression. Living status is not significantly correlated with subjective well-being.

### 3.3 Social support mediates the relation between social participation and subjective well-being (model 1)

**Figure 1:**
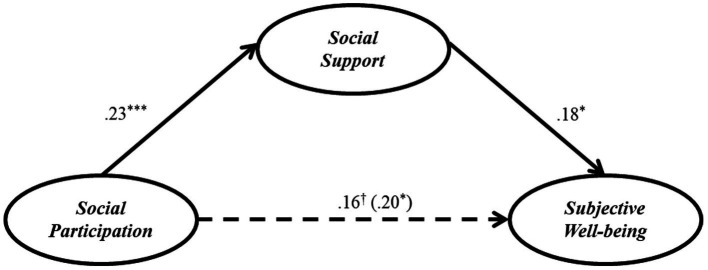
Mediation effect of social support on the relationship between social participation and subjective well-being, controlling for perceived health status, age, gender, and living status. Values represent unstandardized coefficients; Path a: B = 0.23; β = 0.22; *t* = 3.942; SE = 0.06; *p* < 0.001; 95% CI [0.12; 0.35]; Path b: B = 0.18; β = 0.12; *t* = 2.160; SE = 0.09; *p* < 0.05; 95% CI [0.02; 0.35]; Total Effect: B = 0.20; β = 0.12; *t* = 2.292; SE = 0.09; *p* < 0.05; 95% CI [0.03; 0.38]); Direct Effect: B = 0.16; β = 0.10; *t* = 1.783; SE = 0.09; *p* = 0.08; 95% CI [−0.02; 0.34]; Indirect Effect: B = 0.04; β = 0.03; Boot*_SE_* = 0.02; 95% CI [0.002; 0.10].

Figure 1 illustrates the mediation effect of social support on the relationship between social participation and subjective well-being, while controlling for perceived health, age, gender (being female vs. male), and living status (alone vs. with others). The analysis provides evidence that social participation is positively associated with subjective well-being (total effect model: B = 0.20; *β* = 0.12; *t* = 2.292; *SE =* 0.09; *p* < 0.05; 95% CI [0.03; 0.38]). When social support is included as a mediator, this effect is reduced, becoming non-significant (direct effect model: B = 0.16; *β* = 0.10; *t* = 1.783; *SE =* 0.09; *p* = 0.08; 95% CI [−0.02; 0.34]), showing that social support fully mediates this relationship (indirect effect model tested for significance using bias-corrected bootstrap confidence intervals: B = 0.04; *β* = 0.03; Boot*_SE_ =* 0.02; 95% CI [0.002; 0.10]).

### 3.4 Social support mediates the relation between social participation and depression (model 2)

**Figure 2:**
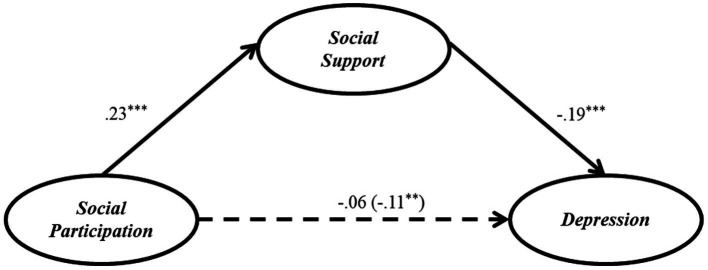
Mediation effect of social support on the relationship between social participation and depression, controlling for perceived health status, age, gender, and living status. Values represent unstandardized coefficients. Path a: B = 0.23; β = 0.22; *t* = 3.942; SE = 0.06; *p* < 0.001; 95% CI [0.12; 0.35]; Path b: B = −0.19; β = −0.26; *t* = −5.130; SE = 0.04; *p* < 0.001; 95% CI [−0.26; −0.12]; Total Effect: B = −0.11; β = −0.14; *t* = −2.643; SE = 0.04; *p* < 0.01; 95% CI [−0.19; −0.03]; Direct Effect: B = −0.06; β = −0.08; *t* = −1.571; SE = 0.04; *p* = 0.12; 95% CI [−0.14; 0.02]; Indirect Effect: B = −0.04; β = 0.06; Boot_SE_ = 0.02; 95% CI [−0.08; −0.02].

Figure 2 illustrates the mediation effect of social support on the relationship between social participation and depression, while controlling for perceived health status, age, gender (being female vs. male), and living status (alone vs. with others). The analysis provides evidence that social participation is associated with reduced depressive symptoms (total effect model: B = −0.11; *β* = −0.14; *t* = −2.643; *SE =* 0.04; *p* < 0.01; 95% CI [−0.19; −0.03]). When social support is included as a mediator, this effect is reduced, becoming non-significant (direct effect model: B = −0.06; *β* = −0.08; *t* = −1.571; *SE =* 0.04; *p* = 0.12; 95% CI [−0.14; 0.02]), showing that social support fully mediates this relationship (indirect effect model tested for significance using bias-corrected bootstrap confidence intervals: B = −0.04; *β* = 0.06; Boot*_SE_ =* 0.02; 95% CI [−0.08; −0.02]).

## 4 Discussion

Despite social participation and social support being consistently identified as predictors of well-being and of physical and mental health among older adults, their interrelations have been less explored, particularly in the context of the restrictions imposed by COVID-19 pandemic. The present study aimed to explore the relationship between social participation, social support, subjective well-being, and depression among older adults residing in a region with low population density, as well as to explore the role of social support mediating the relation between social participation and subjective well-being, and between social participation and depression in the context of restrictions imposed during COVID-19 pandemic.

As expected, according to our hypothesis 1, the results showed that older adults’ social participation during the COVID-19 pandemic was associated with higher social support, higher subjective well-being, and lower levels of depressive symptoms. These findings support previous research identifying social participation as a protective factor for mental health and a promoter of well-being in later life, particularly through the promotion of meaningful social interactions and the strengthening of the sense of belonging (15, 19, 21). Correlation analyses also revealed that self-perceived health status showed moderate associations with subjective well-being and depression, which is consistent with previous studies (e.g., (75, 77). Despite prior studies having identified perceived health as an important determinant of social connectedness in later life, suggesting that better self-rated health is often linked to higher engagement in social activities and stronger support networks (15, 25), including during COVID-19 pandemic [e.g., (68)], our study only revealed weak associations between these variables, suggesting that other sociodemographic variables may have had a greater impact on social participation and social support received during the pandemic period, such as living status. In fact, living status (living alone) was moderately negatively correlated with social participation, in line with previous research highlighting the increased vulnerability of older adults, particularly older women, to psychological distress during periods of social restriction (40, 44). This pattern aligns with existing literature that points to the detrimental effects of social isolation and reduced contact frequency on mental health among older adults, particularly in rural or geographically dispersed areas where opportunities for interaction are more limited (43, 52).

Based on social capital theory and the stress-buffering model, it seems that relational resources may offer some protection against the negative impact of the COVID-19 pandemic on mental health, a period of great adversity, confirming previous studies [e.g., (68)]. In line with these theoretical models and previous studies conducted during normative periods, we would expect social participation to have a direct positive effect on well-being and a negative effect on depression, but that these effects would be amplified by social support, which would play a partial mediating role [e.g., (36, 69)]. However, even though social participation seems to increase subjective well-being and reduce depression, our results demonstrated that social support fully mediated these relationships, only partially confirming our hypotheses 2 and 3. This means that social support accounts the effect of social participation during the COVID-19 pandemic on older adults’ well-being and mental health.

Studying specifically the context of the COVID-19 pandemic, Kwon et al. (70) identified that self-efficacy had a partial mediating effect on the relationship between social support (assessed considering different aspects, such as emotional or tangible) and hopefulness in low-income older adults. However, no published evidence during the COVID-19 pandemic was found reporting the relationship between social participation and subjective well-being or depression being fully mediated by social support in this population group. Therefore, these unexpected findings represent an original contribution in the context analysed. One possible interpretation is that the restrictions on mobility and face-to-face contact, combined with barriers to accessing or using digital resources during the pandemic, may have limited the direct effect of social participation on well-being and mental health due to reduced opportunities for direct emotional or meaningful experiences, making perceived social support the primary pathway (47, 50). This pattern underscores the central importance of the quality and functionality of social relationships in contexts of isolation, beyond the mere frequency of social interactions, particularly in areas with low population density. As Snel et al. (68) observed during the pandemic regarding the protective role of social capital in relation to stress and anxiety, in the present study only social contacts as such (particularly contact with household members and extended family) appeared not to be sufficient to improve well-being or reduce depression. According to social capital theory, this contact may not have been perceived as facilitating mutual aid and sufficient reciprocity during the pandemic, and therefore did not play the more direct protective role that is usually observed. Given the specific characteristics of the older adult population living in low-density areas, it is plausible to hypothesise that a similar full mediation process could also occur in normative periods, outside health crises, particularly among older adults in more vulnerable situations, what should be explored in future studies. However, other potential mediating variables in this relationship between social participation and mental health outcomes in older adults, such as anxiety (71) or psychological resilience [e.g., (72)], should be considered, as previous studies have suggested. On the other hand, potential confounding variables such as personality traits (e.g., neuroticism, extraversion) should be taken into account when interpreting these results and incorporated into future studies. For example, high neuroticism and low extraversion, are known to reduce the likelihood of social participation and simultaneously increase vulnerability to depression [e.g., (73)].

Despite its original contribution and significant implications, our study has some limitations. Firstly, the cross-sectional nature of the study is its main limitation, preventing any causality inferences and directionality of the observed effects. In this context, it should also be considered the possibility that higher subjective well-being and lower depression levels may motivate older adults to engage more socially or perceive more support. Similarly, individuals with better mental health may have and/or develop more easily strong support networks. Secondly, the fact that study was performed in a specific context—COVID-19 lockdown—its generalizability to non-lockdown situations or even to other pandemics/epidemics is limited. However, the purpose was indeed to perform the study in this specific scenario, thus allowing a level of comparability (but not generalizability) to other studies under similar lockdown circumstances. Thirdly, the study is limited by not having included a behavioural measure concerning some variables, as it is the case with “perceived social participation,” which measures the participants subjective assessment of it, rather than actual participation. Nevertheless, it should be noted because the study was done under a lockdown, the application of data collection techniques that would allow assessing actual participation, such as for example, behavioural observation techniques, would be limited by the regulations in place. Still, additional measures could have been used, including for example network size or contact frequency, although these would also represent a perception. A fourth limitation is that, although the sample included a substantial number of participants that were randomly selected, the possibility of survivorship bias should be considered, as older adults with more severe mental health conditions, higher levels of social isolation, or significant physical limitations may have been underrepresented due to non-participation, potentially leading to an underestimation of the true magnitude of social isolation and psychological distress in this population. Finally, the retrospective nature of the data collection, requiring participants to recall their social participation and support during the most critical periods of the COVID-19 pandemic, may have introduced recall bias, as responses could have been influenced by memory inaccuracies, current emotional states, and post-hoc interpretations.

Future studies should seek to examine these relationships with longitudinal data and also enable further comparisons between different situations (with vs. without lockdowns or other situational changes), to enable a better understanding of these and other results. Also, as the data were collected at the end of the pandemic period, participants were asked to recall the most critical phases of the pandemic in order to answer questions about social participation and social support, which may have introduced some recall bias. Furthermore, it will also be important in the future to explore the role that digital technologies might play in maintaining social engagement and promoting social support for older adults in situations where physical contact is restricted. Indeed, studies involving the adult population have highlighted the benefits to well-being resulting from their use during the COVID-19 pandemic [e.g., (74)], and older adults are increasingly using these technologies.

Nevertheless, the results make an important contribution, highlight the importance of making older adults’ social participation relevant and meaningful, reinforcing the role it can play in identifying people who are more vulnerable to depression. Public health policies should be designed to create opportunities for older people to safer participate in social activities during periods of social restrictions. However, these policies must incorporate strategies to strengthen perceived social support networks (of different types and sources), with a focus on the mental health and well-being of the most isolated individuals, particularly in areas with low population density.

## Data Availability

The raw data supporting the conclusions of this article will be made available by the authors, without undue reservation.
